# Nontypeable Haemophilus influenzae Redox Recycling of Protein Thiols Promotes Resistance to Oxidative Killing and Bacterial Survival in Biofilms in a Smoke-Related Infection Model

**DOI:** 10.1128/msphere.00847-21

**Published:** 2022-01-19

**Authors:** Benjamin C. Hunt, Xin Xu, Amit Gaggar, W. Edward Swords

**Affiliations:** a Division of Pulmonary, Allergy and Critical Care Medicine, University of Alabama at Birmingham School of Medicine, Birmingham, Alabama, USA; b Gregory Fleming James Center for Cystic Fibrosis Research, University of Alabama at Birmingham School of Medicine, Birmingham, Alabama, USA; University of Kentucky

**Keywords:** *Haemophilus influenzae*, bacteria, biofilm, pneumonia, smoking-related infection

## Abstract

Smoke exposure is a risk factor for community-acquired pneumonia, which is typically caused by host-adapted airway opportunists like nontypeable Haemophilus influenzae (NTHi). Genomic analyses of NTHi revealed homologs of enzymes with predicted roles in reduction of protein thiols, which can have key roles in oxidant resistance. Using a clinical NTHi isolate (NTHi 7P49H1), we generated isogenic mutants in which homologs of glutathione reductase (open reading frame NTHI 0251), thioredoxin-dependent thiol peroxidase (NTHI 0361), thiol peroxidase (NTHI 0907), thioredoxin reductase (NTHI 1327), and glutaredoxin/peroxiredoxin (NTHI 0705) were insertionally inactivated. Bacterial protein analyses revealed that protein oxidation after hydrogen peroxide treatment was elevated in all the mutant strains. Similarly, each of these mutants was less resistant to oxidative killing than the parental strain; these phenotypes were reversed by genetic complementation. Analysis of biofilm communities formed by the parental and mutant strains showed reduction in overall biofilm thickness and density and significant sensitization of bacteria within the biofilm structure to oxidative killing. Experimental respiratory infection of smoke-exposed mice with NTHi 7P49H1 showed significantly increased bacterial counts compared to control mice. Immunofluorescent staining of lung tissues showed NTHi communities on lung mucosae, interspersed with neutrophil extracellular traps; these bacteria had transcript profiles consistent with NTHi biofilms. In contrast, infection with the panel of NTHi mutants showed a significant decrease in bacterial load. Comparable results were observed in bactericidal assays with neutrophil extracellular traps *in vitro*. Thus, we conclude that thiol-mediated redox homeostasis is a determinant of persistence of NTHi within biofilm communities.

**IMPORTANCE** Chronic bacterial respiratory infections are a significant problem for smoke-exposed individuals, especially those with chronic obstructive pulmonary disease (COPD). These infections often persist despite antibiotic use. Thus, the bacteria remain and contribute to the development of inflammation and other respiratory problems. Respiratory bacteria often form biofilms within the lungs; during growth in a biofilm, their antibiotic and oxidative stress resistance is incredibly heightened. It is well documented that redox homeostasis genes are upregulated during this phase of growth. Many common respiratory pathogens, such as NTHi and Streptococcus pneumoniae, are reliant on scavenging from the host the necessary components they need to maintain these redox systems. This work begins to lay the foundation for exploiting this requirement and thiol redox homeostasis pathways of these bacteria as a therapeutic target for managing chronic respiratory bacterial infections, which are resistant to traditional antibiotic treatments alone.

## INTRODUCTION

Cigarette smoke exposure, be it primary or secondary, imposes a significant economic and health burden in the United States and globally. In the United States alone, a significant proportion of health care expenditures are directed at dealing with smoke-related issues (1). Cigarette smoke has significant impacts on vascular, airway, and immune function, and exposure to cigarette smoke is a significant risk factor for opportunistic infections such as community-acquired pneumonia, chronic bronchitis, and otitis media with bacterial and viral infection (2–4). These infections can exacerbate and further contribute to the development of smoke-associated morbidities.

Smoking is associated with development of chronic bacterial infections which are typically caused by host-adapted opportunists such as nontypeable Haemophilus influenzae (NTHi) (5–9). NTHi is a Gram-negative pathobiont that typically asymptomatically resides in the nasopharynx with little to no overt pathology (10–12). When airway clearance is impaired, NTHi can cause opportunistic infections of the airway mucosal surfaces that include rhinosinusitis, otitis media, and bronchopulmonary infections, which are often chronic or persistent (5, 6, 9, 13) and during which the bacteria are thought to persist within biofilm communities on the airway mucosa (14–19). Biofilms are complex, heterogenous communities that are intransigent to environmental stressors, antibiotics, or host immune effectors largely due to persister subpopulations which are typically found within the biofilm structure (20–22). The cells within bacterial biofilms display unique gene expression profiles, enhanced antimicrobial and oxidative stress resistance, and increased resistance to immune cell clearance compared to planktonically growing bacteria (23–26).

Phagocytes undergo an oxidative burst that culminates in release of reactive oxygen species (ROS), which is a key component of the innate immune response to bacterial infection (27, 28). Thus, maintaining proper redox homeostasis and having mechanisms for counteracting oxidative stress are vital for pathogens to colonize and persist within their host. Bacteria respond to ROS by activating antioxidant defenses, shifting metabolic pathways, and promoting the formation of biofilms (29–31). Glutathione (GSH) is a cysteine-containing thiol tripeptide with important roles in oxidative stress defenses in a wide array of biological systems (32–34). Importantly, Streptococcus pneumoniae and Haemophilus influenzae are reliant on the import of exogenous GSH from the airway environment, where it is abundant (33–35). Peroxiredoxin/glutaredoxin (*pdgX*), thiol reductases, and thioredoxins are thiol metabolic enzymes which are expressed within NTHi biofilms, as well as patient sputa (8, 36, 37).

Analysis of sequenced NTHi genomes revealed a number of homologs of enzymes involved in reduction of oxidized thiols, including predicted glutathione reductase (*gor*, NTHI0251), thioredoxin-dependent thiol peroxidase (*bcp*, NTHI0361), thiol peroxidase (*tpx*, NTHI0907), thioredoxin reductase (*trxB*, NTHI1327) and glutaredoxin/peroxiredoxin (*pdgX*, NTHI0705) (Table 1) (38–40). We used a bacterial genetic approach to generate isogenic mutant strains with predicted impacts on thiol redox metabolism, which were then used to investigate the importance of this pathway in colonization, persistence, and biofilm formation within the airways. We show that disruption of thiol redox homeostasis in NTHi results in significant susceptibility to oxidative stress, susceptibility to bacterial killing within neutrophil extracellular traps (NETs), and defects in bacterial colonization/persistence in experimental respiratory infections in smoke-exposed mice. Based on these results, we conclude that thiol metabolism is an important determinant of NTHi colonization and persistence within biofilm communities.

**TABLE 1:** Gene designations and predicted functions based on homology for genes of interest

Gene (allele)	Predicted function
*gor* (NTHI0251)	Predicted glutathione-disulfide reductase
*bcp* (NTHI0361)	Thioredoxin-dependent thiol peroxidase
*trxB* (NTHI1327)	Thioredoxin-disulfide reductase
*pdgX* (NTHI0705)	Peroxiredoxin-glutaredoxin
*tpx* (NTHI0907)	Thiol peroxidase
*luxS* (NTHI0621)	AI-2 synthesis protein, quorum signaling
*dps* (NTHI1817)	DNA binding ferritin-like protein
*hktE* (NTHI1099)	Catalase

## RESULTS

### Bacterial resistance to oxidative stress.

**FIG 1:**
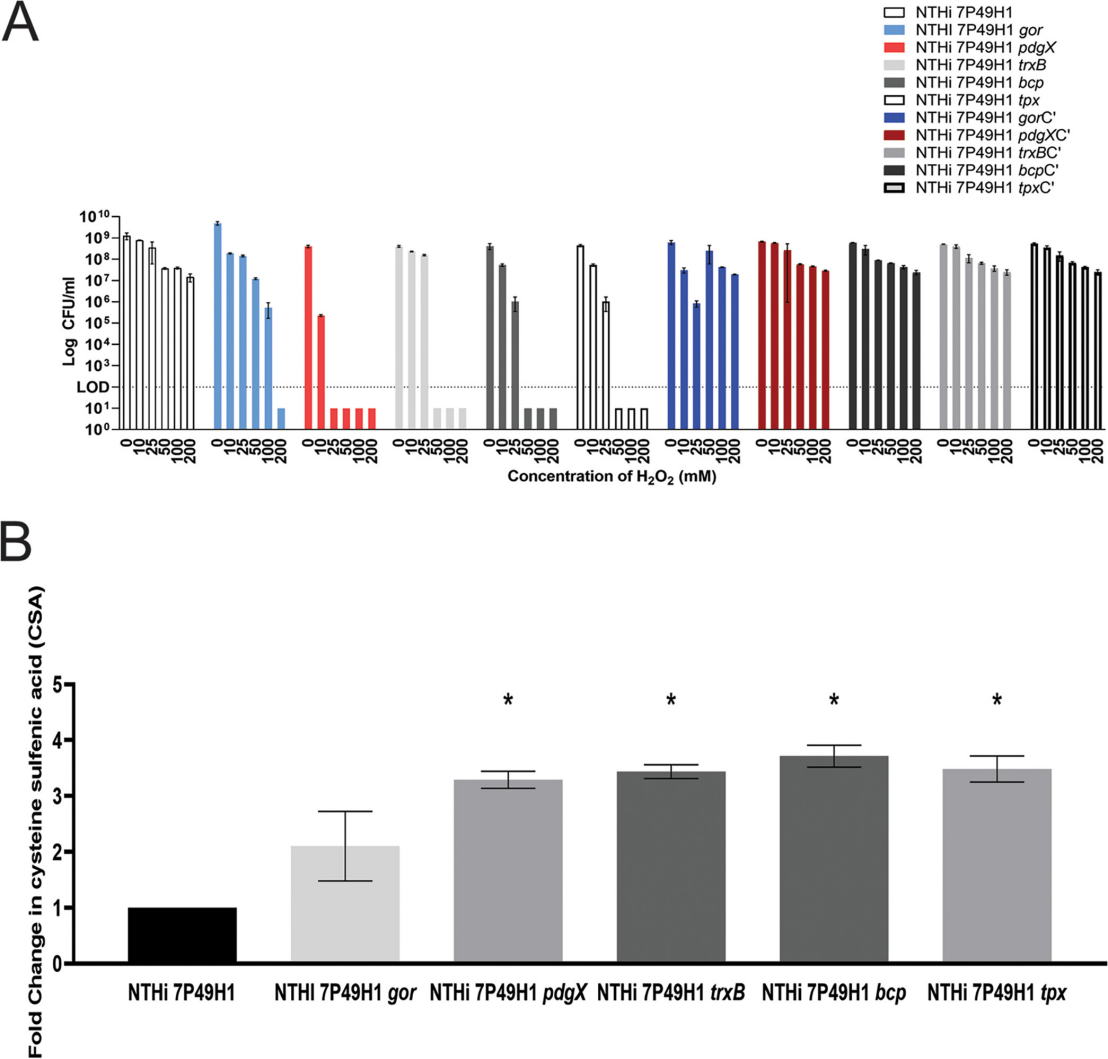
Bacterial susceptibility to various concentrations of H_2_O_2_ and quantification of CSA protein modifications. (A) Oxidative stress and killing of the parent strain, isogenic mutants with mutations affecting thiol redox metabolism, and genetically complemented mutants as determined by viable plate counting postexposure. The dashed line represents the limit of detection (LOD). Data are means and standard errors of the means (SEM) and are representative of three biological replicates. (B) Fold change in CSA protein modifications after exposure to 500 mM hydrogen peroxide as determined by densitometry. Data are means and SD and are representative of three biological replicates. Fold change was analyzed using the Kruskal-Wallis test with Dunn’s multiple comparison. *, *P* < 0.05.

To assess the susceptibility of NTHi 7P49H1 and isogenic mutant strains to oxidative stress, we performed a killing assay in which NTHi biofilms were treated with various concentrations of hydrogen peroxide (H_2_O_2_) for 30 min. Similar to prior experiments with other NTHi strains, minimal killing of NTHi 7P49H1 was observed except at the highest concentrations of hydrogen peroxide (37, 41). In contrast, each of the isogenic mutant strains had significant levels of killing with bacterial counts below the detectable limit when exposed to hydrogen peroxide. There was notable variation between the degree to which individual mutations sensitized NTHi bacteria to oxidative killing, with NTHi 7P49H1 *pdgX* conferring the highest magnitude of defect and with a lesser degree of sensitization being seen with NTHi 7P49H1 *gor*. These results may reflect differences in overall activity or potency of the individual enzymes; these results may also indicate functional redundancy for some of the thiol reduction activity of the individual factors. For the purpose of statistical analyses, all values below the limit of detection were assigned an arbitrary value of 10^1^ CFU. Resistance to oxidants was restored by genetic complementation of the isogenic mutants (Fig. 1A). Comparable results were obtained in parallel experiments with hypochlorous acid (HOCl) as the oxidative stressor (data not shown) and with planktonic bacterial cultures (Fig. S1).

10.1128/mSphere.00847-21.1 FIG S1 Thiol-mediated redox homeostasis has a significant impact on NTHi resistance to oxidative killing. Bacteria were treated with H_2_O_2_ essentially as described in Materials and Methods, and total bacterial populations (planktonic and surface adherent) were measured by plate counting. Counts are means of 4 replicates. Statistical significance was assessed by analysis of variance with a *post hoc* nonparametric *t* test. Download FIG S1, TIF file, 9.7 MB. Copyright © 2022 Hunt et al. 2022 Hunt et al. https://creativecommons.org/licenses/by/4.0/ This content is distributed under the terms of the Creative Commons Attribution 4.0 International license.

### Impact of thiol metabolic factors in protein oxidation.

To further investigate the susceptibility and redox state of the isogenic mutants, we purified oxidized bacterial proteins based on the affinity of cysteine sulfenic acid (CSA) for the nucleophile 1,3-cyclopentanedione (BP1) (42–44). Cysteine sulfenic acids are a reversible posttranslational protein modification that plays a role in redox signaling and oxidative stress-induced protein activity; accumulation of sulfonated proteins is an indication that a cell is under excessive oxidative stress (45, 46). Biotin-linked BP1 was used to purify CSA-modified proteins from bacterial lysates, which were quantified by SDS-PAGE and silver staining. In comparison to the parent strain, thiol redox mutants displayed significantly higher levels of cysteine sulfenic acids and displayed 2- to 3.5-fold-higher changes in cysteine sulfenic acid levels (Fig. 1B). As with the fitness measures in Fig. 1A, it is difficult to reconcile whether differences in impacts of specific factors on protein oxidation or resistance to oxidants are indicative of specific redox activity that is more or less specific for cysteine sulfenic acids or if these factors have overlapping functions. This is indicative of an imbalance in the redox homeostasis of the isogenic mutants and of a heightened sensitivity to oxidative stress. Together, reactive oxygen species killing and the measurement of cysteine sulfenic acids show that disruption of the thiol redox pathway at different steps can similarly sensitize and compromise NTHi’s ability to maintain redox homeostasis.

### Impact of redox homeostasis on NTHi biofilms.

**FIG 2:**
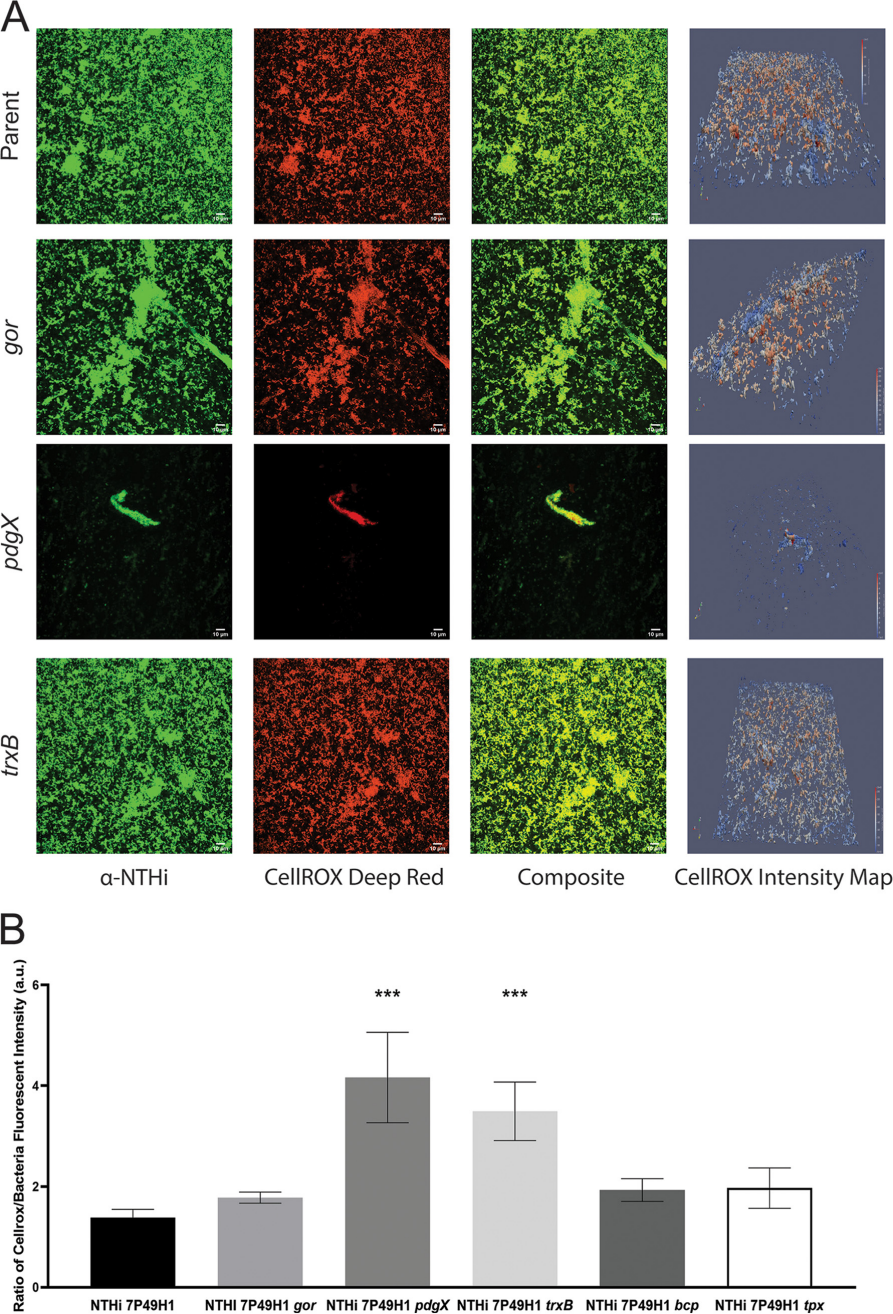
Immunofluorescent imaging and analysis of redox stress of *in vitro* NTHi biofilms. (A) Bacteria were cultured as static biofilms for 24 h, treated with 500 mM hydrogen peroxide, and stained with anti-NTHi antibodies conjugated to Alexa Fluor 488 (green), while bacterial oxidative stress was visualized using CellROX Deep Red (red). Fluorescent pixel intensity maps of the CellROX channel were generated using BiofilmQ. Images were taken at ×60 magnification. Bars, 10 μm. (B) Mean pixel intensity of the CellROX channel was quantified using BiofilmQ software. Data are means and SD (*n* = 5). Statistical significance was assessed by Kruskal-Wallis one-way ANOVA with a *post hoc* nonparametric *t* test. ***, *P* < 0.0005.

To further investigate the redox homeostasis and oxidant resistance of our isogenic mutant biofilms when exposed to oxidative stress, we utilized confocal scanning laser microscopy alongside BiofilmQ to visualize and quantify oxidative stress within the biofilm. Using confocal imaging, we generated vertical Z-series images of NTHi biofilms after exposure to 500 mM hydrogen peroxide, with the objectives of defining any effects on biofilm formation/maturation and identifying and localizing bacterial subpopulations differentially impacted by oxidative stress. NTHi formed thick communities with three-dimensional height and structure, which were maintained after exposure to oxidative stress. In contrast, NTHi 7P49H1 *pdgX* biofilms were largely diminished in three-dimensional structure following peroxide treatment, consistent with reduced resistance (Fig. 2A). Importantly, untreated biofilms for the mutant bacterial strains had no changes in biofilm density (Fig. 2), and the NTHi mutant strains had various levels of sensitization to oxidants in the planktonic as well as the biofilm phase of growth (Fig. S1). It is also important to note that no differences in biofilm biomass were observed under conditions without oxidative stress (Fig. S2).

10.1128/mSphere.00847-21.2 FIG S2 Crystal violet staining of 24-h NTHi biofilms. Crystal violet absorbance was measured at an optical density of 540 nm. Data are means and SD (*n* = 3). Statistical significance was assessed by a nonparametric *t* test. Download FIG S2, TIF file, 7.1 MB. Copyright © 2022 Hunt et al. 2022 Hunt et al. https://creativecommons.org/licenses/by/4.0/ This content is distributed under the terms of the Creative Commons Attribution 4.0 International license.

The areas of the biofilm closest to the substrata and the internal areas of mature tower structures displayed the highest intensity of CellROX fluorescent signal, and thus, these areas were presumably under the most oxidative stress (Fig. 2A and Fig. S3). Additionally, when utilizing the BiofilmQ software to quantitate the mean pixel intensity of the CellROX fluorescent signal, we found that the isogenic mutants displayed heightened mean pixel intensities. Of the isogenic mutant strains, NTHi 7P49H1 *pdgX* and NTHi 7P49H1 *trxB* showed significantly higher mean pixel intensities of CellROX than the parent strain. The NTHi 7P49H1 *pdgX* isogenic mutant displayed the highest mean intensity, nearly double the mean for the parent strain (Fig. 2B). Additionally, when measuring the ratio of the CellROX pixel intensity to the pixel intensity of NTHi, we saw that this relationship was maintained, with thiol redox pathway mutants showing higher ratios than the parent strain (Fig. S3). This indicates that the isogenic mutant biofilms are inherently more susceptible to oxidative stress than the parent strain, particularly the biofilm formed by NTHi 7P49H1 *pdgX*. It is particularly noteworthy that *pdgX* has been reported to be expressed at higher levels in NTHi biofilms *in vitro* (8, 37, 47), as well as in experimental infections (37) and sputa from patients with chronic obstructive pulmonary disease (COPD) who have chronic H. influenzae infections (8).

10.1128/mSphere.00847-21.3 FIG S3 Immunofluorescent imaging and analysis of redox stress of NTHi 7P49H1 *bcp* and 7P49H1 *tpx in vitro* biofilms. (A) Immunofluorescent staining of biofilms exposed to 500 mM hydrogen peroxide. Bacteria were stained with anti-NTHi rabbit polyclonal sera and secondary Alexa Fluor 488 antibody conjugate (green), while bacterial oxidative stress was visualized using CellROX Deep Red (red). Fluorescent pixel intensity maps of the CellROX channel were generated using BiofilmQ. Images were taken at ×60 magnification. Bars, 10 μm. (B) Ratio of mean pixel intensity of CellROX to mean pixel intensity of NTHi. Data are means and SD (*n* = 5). Statistical significance was assessed by a Mann-Whitney nonparametric *t* test. Download FIG S3, TIF file, 23.1 MB. Copyright © 2022 Hunt et al. 2022 Hunt et al. https://creativecommons.org/licenses/by/4.0/ This content is distributed under the terms of the Creative Commons Attribution 4.0 International license.

### Bacterial colonization and persistence in mouse lungs.

**FIG 3:**
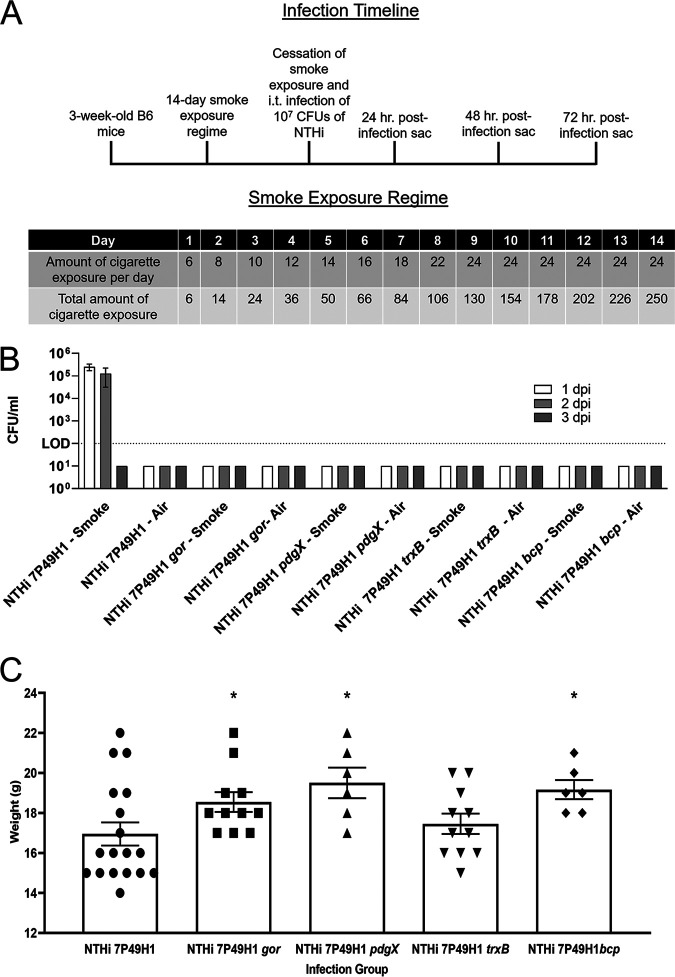
Bacterial respiratory infection of smoke-exposed mice and air controls. (A) Schematic detailing infection timeline and smoke exposure regimen for mouse cohorts. (B) Persistence in smoke-exposed and air control mice as determined by viable plate counting of lung homogenate posteuthanasia. The dotted line indicates the LOD. dpi, days postinfection. Data are means and SEM (*n* = 6 to 18). (C) Weights of mice 24 h postinfection. All weight were compared to those of the parent strain-infected animal group. Data are means and SEM (*n* = 6 to 18) and are representative of three independent biological replicates. Statistical significance was assessed by the Mann-Whitney nonparametric *t* test. *, *P* < 0.05.

To define impact of disruption of redox homeostasis on bacterial colonization/persistence *in vivo*, we performed infection studies using smoke-exposed mice. Mice (C57/BL6) were treated daily with cigarette smoke as outlined in Materials and Methods and Fig. 3A. Bacteria were recovered from the lungs of smoke-exposed mice infected with NTHi 7P49H1 for up to 48 h postinfection; this was in contrast to untreated control mice, which cleared infection. Each of the thiol redox mutants tested was unable to establish a successful infection in the susceptible smoke-exposed mouse lung, showing no detectable amounts of NTHi at any time postinfection (Fig. 3B). Cigarette smoke alone can reduce the weight of mice; however, when mice are infected with NTHi, there is a synergist effect, and animals lose significantly more weight in addition to the loss induced by smoke (Fig. S3). However, mice infected with isogenic thiol redox pathway mutants had significantly less weight loss over the course of infection, indicating overall less severe disease.

### NTHi forms biofilms within smoke-exposed mouse lungs.

**FIG 4:**
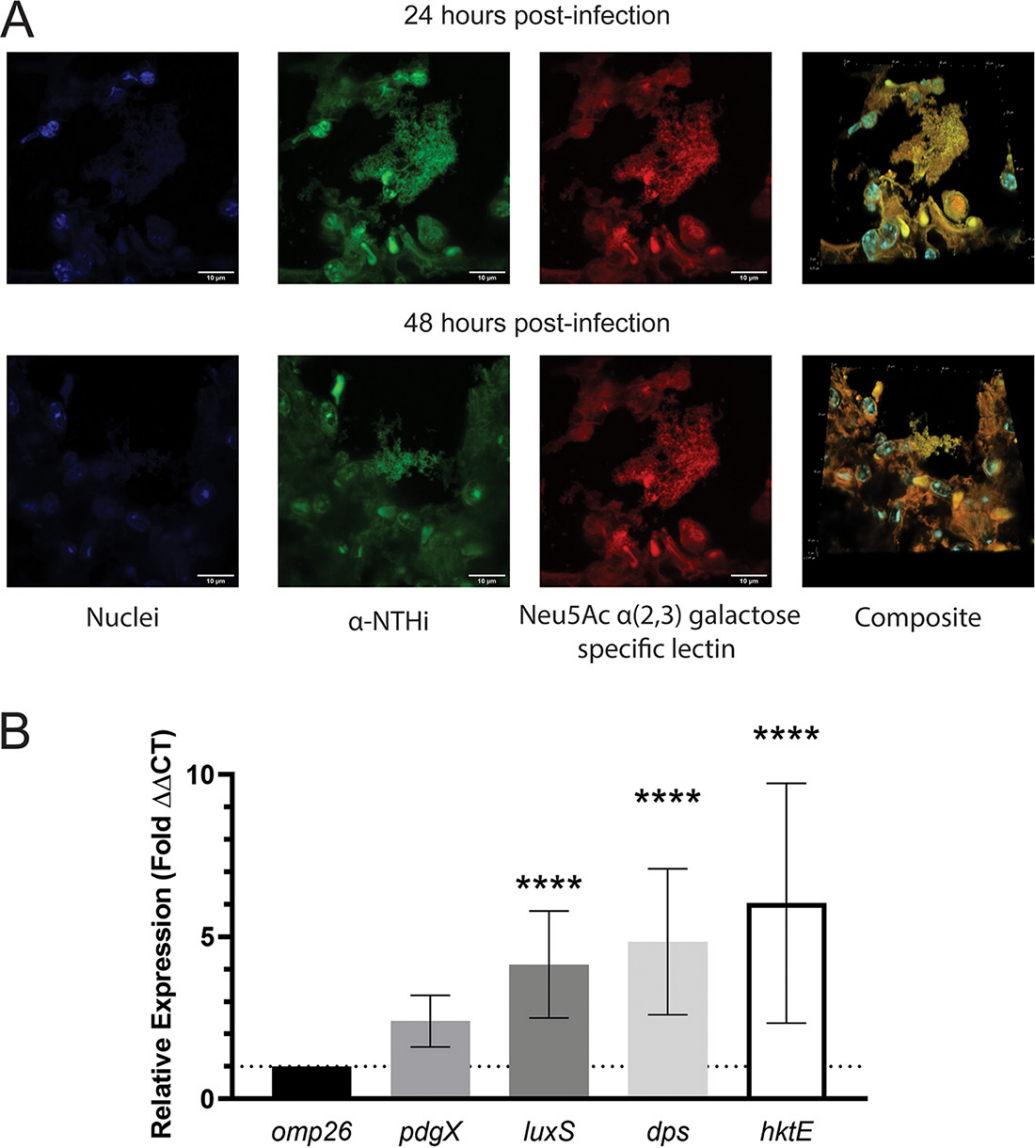
Immunofluorescent imaging and RT-qPCR analysis of NTHi biofilms in the smoke-exposed murine lung. (A) Immunofluorescent staining of NTHi infected mouse lung sections. Lung sections are representative of 24 and 48 h postinfection. Nuclei were stained with DAPI (blue), and bacteria were stained with anti-NTHi rabbit polyclonal sera and secondary Alexa Fluor 488 antibody conjugate (green). NTHi biofilm components were stained using the Maackia amurensis lectin conjugated to Texas Red, which is specific for terminal Neu5Ac α(2,3)galactose (red). Images were taken at ×90 magnification. Bars, 10 μm. (B) RT-qPCR analysis of mRNA isolated from infected mouse lung of biofilm associated genes, in comparison to *omp26* housekeeping gene. Samples were collected 24 h postinfection. All samples were run in triplicate. Data are means and SD (*n* = 4 to 6). Statistical significance was assessed by ANOVA and a *post hoc* test of significance essentially as for the preceding figures. ****, *P* < 0.00005.

Using confocal scanning laser microscopy revealed that in the airways of smoke-exposed mice infected with the parent strain multicellular NTHi communities can be detected at 24 and 48 h postinfection (Fig. 4A). These multicellular communities are absent in the airways of animals infected with isogenic thiol redox pathway mutants. To confirm that these multicellular communities found within the airways were NTHi biofilms, we stained the tissue sections with fluor-conjugated lectins that would bind specific linkages within the NTHi biofilm extracellular matrix. Doing so revealed that our aggregates of NTHi overlap the staining for the biofilm extracellular matrix, thus showing that these communities are indeed imbedded in a biofilm-like structure (Fig. 4A). To ask if NTHi bacteria in the lung had gene expression profiles consistent with NTHi biofilms, we measured bacterial transcripts for genes known be expressed either exclusively or at higher levels in biofilm (*pdgX*, *luxS*, and *dps*). Notably, all of these factors had increased expression *in vivo* during our infection experiments (Fig. 4B). Combined with the confocal imaging, the expression of biofilm-associated genes further confirms that these structures are indeed NTHi biofilms forming within the lungs of susceptible smoke-exposed mice.

### Bacterial persistence within neutrophil extracellular traps.

**FIG 5:**
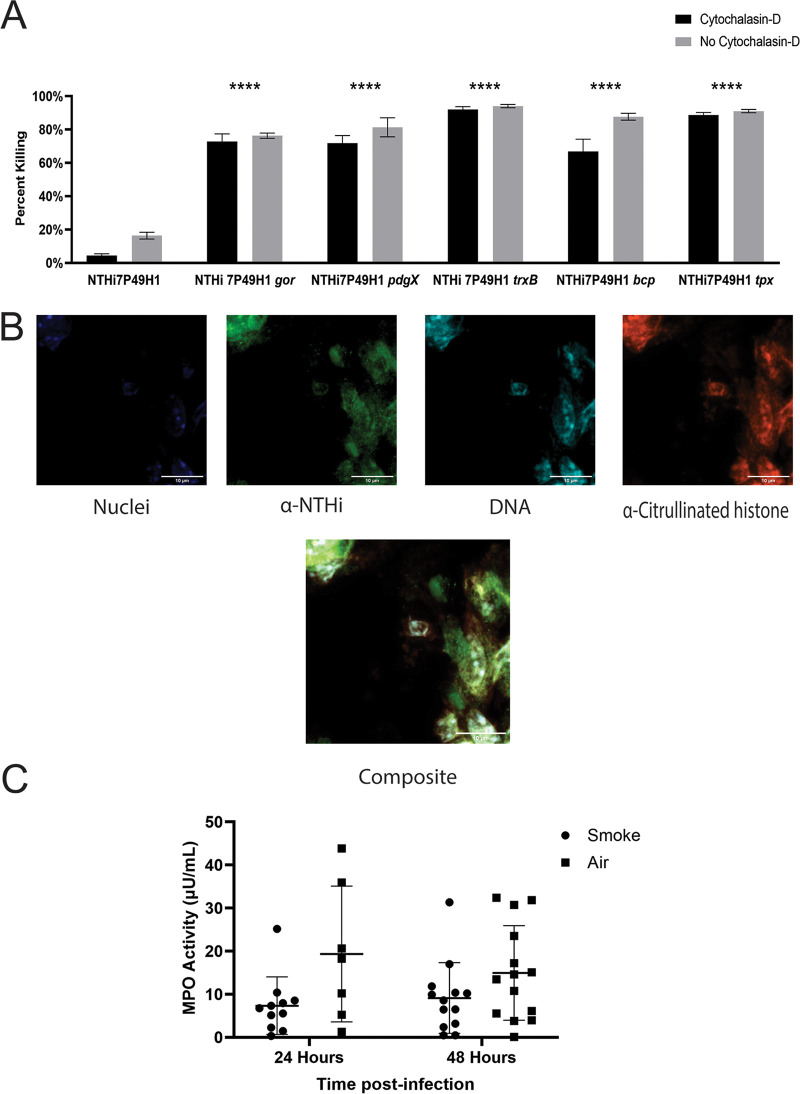
NET killing assay and fluorescent staining for NETs in infected mouse lungs. (A) HL60 immortalized promyeloid cells were differentiated for 5 to 6 days, seeded in 24-well plates, and activated with 25 nM phorbol myristate acetate, after which 20 μM cytochalasin D was added to prevent killing via phagocytosis. Bacteria were added at an MOI of 10. Bacterial killing was expressed as a percentage of the counts obtained from control wells. Data are means and SEM (*n* = 3). All statistical comparisons were to the matched treatment of the parent strain. ****, *P* < 0.00005. (B) Immunofluorescent staining and confocal imaging of infected mouse lung sections. Nuclei were stained with DAPI (blue), bacteria were stained with anti-NTHi rabbit polyclonal sera and secondary Alexa Fluor 488 antibody conjugate (green), and NETs were stained using anti-histone H3 citrulline R2, R8, and R17 (red) and propidium iodide (cyan). These confocal images are representative images of mouse lungs 48 h postinfection. Images were taken at ×90 magnification. Bars, 10 μm. (C) MPO activity in lung homogenate. Data are means and SD (*n* = 12 to 18). Statistical significance was assessed by the Mann-Whitney nonparametric *t* test.

Prior work from our group has shown that resistance to oxidants is central NTHi survival and growth within neutrophil extracellular traps and thus important for establishing a chronic infection (26, 37, 48). To investigate the susceptibility of our mutant strains to killing via neutrophil extracellular traps, we used differentiated HL60 immortalized monocytes activated with phorbol myristate acetate and inhibited phagocytosis using cytochalasin D. NTHi 7P49H1 was highly resistant to killing via neutrophil extracellular traps, as we have reported for other H. influenzae strains (26, 37, 49). In contrast, all of bacterial thiol redox mutant strains were significantly more susceptible to killing (Fig. 5A). Similarly, confocal analysis of lung tissue sections for H. influenzae bacteria within NET structures (DNA and citrullinated histone H3, both components of neutrophil extracellular traps) showed abundant NTHi multicellular communities surrounded by neutrophil extracellular traps in the airways of smoke-exposed mice 48 h postinfection (Fig. 5B). No such bacterial communities were observed in mice infected with NTHi mutants. Additionally, using a myeloperoxidase activity assay to measure myeloperoxidase (MPO) activity within the lung, we saw similar levels of activity in both air- and smoke-exposed NTHi-infected animals (Fig. 5C). Based on these results, we conclude that maintenance of thiol redox pathway homeostasis is a determinant of NTHi survival within NETs *in vivo*.

## DISCUSSION

NTHi is an opportunistic pathogen that is highly adapted to colonize and persist within the human respiratory tract; in fact, many of the virulence-related attributes of NTHi impact asymptomatic carriage within the nasopharynx and upper airways in addition to disease presentations (50, 51). For example, it is now well established that NTHi bacteria persist *in vivo* within multicellular biofilm communities on and within the airway epithelia (15, 47, 50–53), and many of the determinants of NTHi biofilm formation have been shown to have significant roles in bacterial colonization and persistence *in vivo* during NTHi opportunistic infections (50, 53–58). In this study, we observed that NTHi colonization and persistence within the lung are significantly enhanced by short-term smoke exposure, which roughly approximates acute or second-hand smoke. This is consistent with prior work showing increased susceptibility to NTHi infection for mice following chronic smoke exposure; we recently extended these findings to show increased NTHi colonization/persistence and inflammatory exacerbation symptoms in ferrets following chronic smoke exposure (47). As in our ferret infection studies, our results in this study support the conclusion that persistent NTHi bacteria in smoke-exposed mice survive within biofilm communities; this is validated by biofilm-related bacterial changes that include visualization of multicellular aggregates in the airway lumen, encased within a sialylated polysaccharide matrix consistent with that described for H. influenzae (47, 56, 59), and gene expression profiles that are consistent with H. influenzae in the biofilm mode of growth (59, 60). Importantly, these findings may have significance beyond the well-established connection between COPD and H. influenzae infection and could potentially indicate a role for H. influenzae biofilms in pulmonary infections such as community-acquired pneumonia, which can be significantly impacted by short-term or indirect smoke exposure.

Our observation of NTHi bacteria within neutrophil extracellular traps also merits comment. Contrary to roles for NETs in facilitating clearance of some microbes *in vivo*, we have consistently observed that NTHi bacteria survive and persist within NETs (26, 37, 49). Importantly, bacterial surface moieties that promote biofilm formation/maturation also have significant roles in NTHi persistence within NETs (26), which may indicate a central role for NTHi biofilms in long-term survival within NETs *in vivo*. More recently, work from our laboratory also showed that NTHi genes with predicted roles in resistance to oxidative stress have increased expression in biofilms (37, 61) and, moreover, that many of these factors are important to NTHi survival within biofilms *in vivo* and within *in vitro* models for NETs (37). The results from this study show that maintenance of NTHi redox homeostasis via reduction of oxidized protein thiols is an important determinant of bacterial survival within NETs and colonization/persistence *in vivo* and, importantly, that the bacterial populations within the biofilm are affected by mutations that ablate thiol metabolic recycling. These findings indicate that bacterial persister subpopulations may be acutely affected by disruption of redox homeostasis, and given that these are thought to be the populations with the highest level of resistance against antimicrobials, these findings may highlight a specific way to target resistant NTHi subpopulations. It is notable that the primary biothiol utilized by H. influenzae (and pneumococcus as well) is glutathione, which is scavenged from the airway surface fluid and mucus (34, 35). We also note that the transcript levels for the NTHi peroxiredoxin/glutaredoxin *pdgX* were increased in tissue homogenates from infected mice, which is consistent with prior work showing that this factor in particular is increased in NTHi biofilms and patient sputa (8, 37). Also of note, our data clearly indicate that mutants lacking *pdgX* are particularly impaired for survival of oxidative stress within biofilms. It is thus reasonable to speculate that therapeutic impairment of glutathione uptake and/or metabolism by H. influenzae could specifically target persistent bacterial populations within biofilms; such targeted therapeutics alone or in conjunction with existing antibiotic therapies could have a significant impact on a range of prevalent and highly costly infections.

## MATERIALS AND METHODS

### Bacteria and culture methods.

NTHi 7P49H1 was isolated from sputum cultures from a patient with chronic obstructive pulmonary disease (62). NTHi bacteria were cultured at 37°C on brain heart infusion agar (Difco, NJ, USA) supplemented with NAD (10 μg/mL; Sigma) and hemin (10 μg/mL; ICN Biomedicals); here, this medium formulation is referred to as supplemented BHI (sBHI). Bacteria were harvested from the surfaces of overnight culture plates and resuspended in phosphate-buffered saline (PBS) to the desired optical density to generate inocula.

### Generation and complementation of thiol redox pathway mutants.

To generate the thiol redox pathway mutants, a fragment containing the open reading frame of the gene of interest with at least 500 bp of upstream and downstream flanking DNA was amplified using the appropriate primers (Table 2). The resulting amplicon was cloned into pCR2.1 (Invitrogen, Waltham, MA) at the HindIII and XhoI restriction sites following the manufacturer’s instructions to generate the plasmids pCR2.1*gor*, pCR2.1*bcp*, pCR2.1*trxB*, pCR2.1*pdgX*, and pCR2.1*tpx*; the identities of DNA inserts in all plasmids were confirmed by sequence analysis. XhoI restriction sites were introduced within the coding sequence of each open reading frame using primers listed in Table 2; antibiotic resistance cassettes were excised from pCMr or pSpecr with XhoI and inserted into target genes by DNA ligation to generate plasmids pCR2.1*gor*::spec, pCR2.1*bcp*::cm, pCR2.1*trxB*::spec, pCR2.1*pdgX*::cm, and pCR2.1*tpx*::spec (Table 2). Plasmid constructs were confirmed by sequence analysis and PCR amplification of the gene of interest. For generation of isogenic mutant NTHi strains, linearized DNA containing the antibiotic-tagged null alleles were introduced into NTHi 7P49H1 using a colony transformation method we described previously (52, 54, 63). Transformants were recovered by plating onto sBHI agar plates containing the appropriate antibiotic and incubation at 37°C and 5% CO_2_ for 1 to 2 days. Transformants were isolated, and null mutants were confirmed via PCR and sequence analysis.

**TABLE 2:** Primers and plasmids used in this study

Designation	Sequence or description	Source or reference
Primers		This study
NTHi *gor* flanking F	CTAGAGATCTTTATTTTTTATATGCCAAGATTTCG	This study
NTHi *gor* kof *Xho*I	CTCGAGAATGCAGATGGTTCACTTACTGTAA	This study
NTHi *gor* flanking R	CTAGAGATCTACGCTAATTGGTTATAAGATTAATT	This study
NTHi *gor* kor *Xho*I	CTCGAGAATTTCAGATGGGGTAGAATTGGT	This study
NTHi *bcp* flanking F	CTAGAGATCTATGGTACAACTTTTTGATTT	This study
NTHi *bcp* kof *Xho*I	CTCGAGAGTTGCGGAGCAATTTGGCG	This study
NTHi *bcp* flanking R	CTAAAACGCGACTGTAAGATCTCTAG	This study
NTHi *bcp* kor *Xho*I	CTCGAGTCAGGATCAGAAAGCAATGTGAAAT	This study
NTHi *trxB* flanking F	TAAAATTCCAACAAGCATTA	This study
NTHi *trxB* kof *Xho*I	CTCGAGAATTGCAAGTACAGTGCATTTAATCCAC	This study
NTHi *trxB* flanking R	AAGCCTGCAGCCCAG	This study
NTHi *trxB* kor *Xho*I	CTCGAGAAGTAAAGGGCTTCTTCCACCG	This study
NTHi *pdgX* flanking F	TTATCATATTTAATAACTTCTTTGT	This study
NTHi *pdgX* xof *Xho*I	CTCGAGAATTCACCATTACCATCTGGAATGAA	This study
NTHi *pdgX* flanking R	AGTAACATTTTCTCGTTGAA	This study
NTHi *pdgX* xor *Xho*I	CTCGAGAAGGCATGGGTATGTTAGTTGGT	This study
NTHi *tpx* flanking F	ACGAACGTTTTCCTGAGCGTG	This study
NTHi *tpx* xof *Xho*I	CTCGAGGCCCTTTGCTCAAGCTCGTT	This study
NTHi *tpx* flanking R	TATACTCGCTCTACAACCGCT	This study
NTHi *tpx* xor *Xho*I	CTCGAGGCTGAAATACAAAGCACAACAGT	This study
SpecF *Xho*I	CTCGAGAGAATTCGGATCCCGGGATATCT	This study
SpecR *Xho*I	CTCGAGGAATTCCCGGGATCGGATCC	This study
CmF *Xho*I	CTCGAGATGGAGAAAAAAATCACTGGATATACC	This study
CmR *Xho*I	CTCGAGTTACGCCCCGCCCTG	This study

Plasmids		This study
pCR2.1	Cloning vector	Invitrogen
pCR2.1*gor*	*gor* clone	This study
pCR2.1*bcp*	*bcp* clone	This study
pCR2.1*trxB*	*trxB* clone	This study
pCR2.1*pdgX*	*pdgX* clone	This study
pCR2.1*tpx*	*tpx* clone	This study
pCMr	Plasmid containing chloramphenicol resistance cassette used to generate mutants	60
pSpec	Plasmid containing spectinomycin resistance cassette used to generate mutants	61
pCR2.1*gor*::spec	*gor* null mutant	This study
pCR2.1*bcp*::cm	*bcp* null mutant	This study
pCR2.1*trxB*::spec	*trxB* null mutant	This study
pCR2.1*pdgX*::cm	*pdgX* null mutant	This study
pCR2.1*tpx*::spec	*tpx* null mutant	This study
pACYC184	Cloning vector used to complement mutants	73
pACYC184*gor*	*gor* complementation	This study
pACYC184*bcp*	*bcp* complementation	This study
pACYC184*trxB*	*trxB* complementation	This study
pACYC184*pdgX*	*pdgX* complementation	This study
pACYC184*tpx*	*tpx* complementation	This study

For genetic complementation, each gene was cloned into pACYC184 to generate pACYCY184*gor*, pACYCY184*bcp*, pACYCY184*trxB*, pACYCY184*pdgX*, and pACYCY184*tpx;* all plasmids were confirmed by sequence analysis. NTHi was grown overnight on sBHI agar plates, after which the plasmids were introduced into the appropriate isogenic mutant strain by electroporation according to established methods (64). NTHi cells were washed and electroporated at 2.5 kV using Bio-Rad Micropulser (Bio-Rad, Hercules, CA). After 1 to 2 h of recovery in prewarmed sBHI, bacteria were plated on the appropriate antibiotic medium. Sequence analysis was also performed to ensure the plasmid had been transformed.

### Measurement of biofilm-associated bacterial resistance to oxidants.

Bacteria were suspended in sBHI, seeded at a concentration of ∼10^8^ CFU/mL into a 24-well dish, and cultured at 37°C and 5% CO_2_ for 24 h, after which growth medium was aspirated and replaced with PBS containing various concentrations of hydrogen peroxide, as indicated in the figure legends. Bacteria were exposed to oxidant for 30 min, after which surface-adherent bacteria were gently washed with PBS three times. Bacterial biofilms were then scraped off the bottom of the well, serially diluted, and plated on sBHI for plate counting.

### Static biofilm assay.

Flat-bottom 24-well plates were seeded with 10^8^ CFU/mL of NTHi in a 1-mL volume. The parent strain and each thiol redox pathway mutant were seeded in triplicate. Biofilms were grown for 24 h at 37°C with 5% CO_2_ without shaking. After incubation, plates were removed and medium was aspirated from the well. The wells were then washed twice with 1 mL of water to remove planktonic bacteria. Plates were then dried for 30 min at 37°C. Wells were then stained with 1 mL of 0.1% crystal violet solution for 30 min. After staining, wells were washed twice again with 1 mL of water. Care was taken to be gentle with the washing to ensure that excess biofilm was not dislodged. Next, 1 mL of 30% acetic acid was added to each well, and the plate was incubated on a shaker for 10 min to solubilize the crystal violet stain. Absorbance was read at an optical density of 540 nm.

### Quantification of cysteine sulfenic acid oxidation.

Bacteria were resuspended in PBS to ∼10^8^ CFU/mL; bacterial density was confirmed by plate counts. Bacteria were then centrifuged, and the supernatant was removed and replaced with a 500 mM hydrogen peroxide solution. The mixture was incubated for 30 min at 37°C, after which the supernatant was removed and the pellet was washed with PBS. Bacteria were lysed enzymatically using a lysis buffer (50 mM Tris [pH 8.0], 10% glycerol, 0.1% Triton X-100, and 100 mg/mL lysozyme) with simultaneous labeling for cysteine sulfenic acids (CSAs) with 1 mM biotin-1,3-cyclopentanedione (BP1) (Kerafast, Boston, MA, USA). Lysis buffer was prepared fresh before use, and BP1 was added immediately prior to use. Samples were lysed and labeled for CSAs for 1 h at 37°C. After lysis and biotin labeling of CSAs, the CSA-modified proteins were isolated using Bio Capturem streptavidin miniprep columns (TaKaRa, Shiga, Japan) following the manufacturer’s instructions. Next, samples were run on an 4 to 20% gradient SDS-PAGE gel, stained using the Pierce silver stain kit, and imaged. Relative CSA protein modifications was determined via densitometry using ImageJ Fiji (26).

### Cigarette smoke exposure and mouse infections.

Mice (C57BL/6J) were acquired from Jackson Laboratory (Bar Harbor, ME, USA) and randomly assigned to an experimental group, either the one exposed to whole cigarette smoke or the ambient air control. For each biological replicate, each experimental group consisted of 6 mice. This strain of mice was used because they are susceptible to cigarette smoke and have been used in a variety of smoke exposure experiments (65–68). Smoke group mice were placed in a whole-body exposure chamber and exposed to smoke from 3R4F research cigarettes (Louisville, KY, USA), twice daily for a period of 14 days. Animals were given half their daily allotted number of cigarettes in the first exposure of the day, after which animals were allowed a 2-h rest period, and then finally animals were given the remaining half of their daily allotted number of cigarettes. Animals began smoke exposure at a minimum of 6 cigarettes per day, with the total number of cigarettes increasing by 2 per day until reaching 24 total per day and then remaining constant for the remainder of the regimen. This regimen was selected to simulate acute-smoke-exposure models, which have been shown to induce significant inflammatory, genetic, and injury responses in the lung (38–40, 69, 70). Animals were monitored continuously during smoke exposure. Cigarette smoke was generated by an automated cigarette smoke generator (SCIREQ; InExpose model), with a 24-cigarette carousel. SCIREQ filters were monitored and weighed to measure total particulate-matter exposure for comparison to other murine smoke models. Animals exposed to smoke were housed separately from the air control groups. After completing the smoke exposure regimen, mice were intratracheally infected with 10^7^ CFU of NTHi or vehicle control (PBS). Animals were euthanized 24, 48, and 72 h postinfection. Lung tissue was homogenized using the TissueLyser II (85300; Qiagen, Hilden, Germany). Samples were homogenized at 30 Hz for 3 min. All animal and infection procedures were performed according to AVMA laboratory standard procedures and were reviewed and approved by the UAB Institutional Animal Care and Use Committee.

### Measurement of bacterial killing by neutrophil extracellular traps.

Derivation and measurement of NTHi killing by neutrophil extracellular traps was essentially as described in previous studies (26, 37, 49). HL60 monocyte cells were cultured in RPMI 1640 (Thermo Fisher, MA, USA) with 10% fetal bovine serum (FBS), differentiated for 5 to 6 days in RPMI containing 0.8% dimethyl formamide, and then collected by centrifugation. Cells (∼10^6^/well) were seeded into the wells of a 24-well dish and activated with 25 nM phorbol myristate acetate (PMA) (Sigma-Aldrich, MO, USA) for 10 min. NET formation was confirmed via microscopy. Cell culture medium, with or without 20 μM cytochalasin D, was then added, and cells were incubated for 15 min. Bacterial strains were then added at a multiplicity of infection (MOI) of 10 in triplicate, incubated for 30 min, scraped, serially diluted, and plated to enumerate bacterial counts. Bacterial killing was expressed as a percentage of counts obtained from control wells with no HL60 cells. NET versus phagocytic killing was assessed by comparison of wells with cytochalasin D to those without.

### Myeloperoxidase activity.

The MPO activity in mouse lung homogenate was measured using the MPO activity assay kit (ab111749; Abcam, Cambridge, UK) according to the manufacturer’s instructions. Briefly, tissue homogenates were flash frozen in liquid nitrogen and centrifuged to remove insoluble material; the supernatant was collected, and MPO activity was assessed by time course measurements of linear fluorescence absorbance/emission every 2 min for a period of 30 min using a Tecan Spark plate reader (Tecan, Männedorf, Switzerland). MPO activity is in picomoles per minute per milliliter or microunits per milliliter.

### Immunofluorescent staining and confocal laser scanning microscopy.

Mouse lung tissue sections were sectioned (Thermo Fisher CryoStar NX70 cryostat, 5 μm/section) and fixed onto glass slides. NTHi bacteria were stained using polyclonal rabbit anti-H. influenzae sera and goat anti-rabbit IgG Alexa Fluor 488 secondary antibody conjugate (Thermo Fisher). Coverslips were mounted using Prolong Gold antifade reagent with DAPI (4′,6-diamidino-2-phenylindole; Thermo Fisher, Waltham, MA). Confocal laser scanning microscopic analyses were performed using a Nikon A1R TE2000 inverted microscope (Nikon, Tokyo, Japan). To measure oxidative conditions in biofilms, CellROX Deep Red (Thermo Fisher, Waltham, MA) was used to image and measure oxidative stress in bacterial biofilms, following the manufacturer’s instructions. DNA was imaged using propidium iodide (pseudocolored to cyan for improved visibility). Citrullinated histone was visualized using mouse monoclonal antibody and relevant secondary antibody fluorescent conjugate (Invitrogen). Pixel intensity maps and quantification of biofilm images were performed using BiofilmQ software (71). Images were segmented using the semimanual Otsu threshold method. Terminal Neu5Ac α(2,3)galactose found within the NTHi biofilm matrix (47, 56, 59) was stained utilizing Texas Red-conjugated Maackia amurensis lectin (EY Laboratories, San Mateo, CA). Representative images were created using Fiji imaging analysis software (72).

### Transcript quantification using qRT-PCR.

Bacterial RNA was extracted using the Monarch total RNA miniprep kit (New England Biolabs, Ipswich, MA) following the manufacture’s guidelines. RT-qPCR was performed using the Applied-Biosystems 7500 System, and oligonucleotide probes specific for *pdgX*, *luxS*, *dps*, *hktE*, and *omp26* (Table 3). The *omp*26 transcript was chosen as an endogenous control because its expression does not vary between planktonic and biofilm modes of growth (44). The NEB Luna universal reaction mix was used according to the manufacturer’s directions for cycling conditions (New England Biolabs, Ipswich, MA). All samples were run in duplicate. Transcript measures were normalized relative to *omp26* levels from the same sample. Relative quantification of gene expression was determined using the comparative cycle threshold (*C_T_*) method (2^ΔΔ^*^CT^*).

**TABLE 3:** RT-qPCR primers and probes used for analysis of biofilm expression genes

Target gene	Primer or probe	Sequence (5′ to 3′)^*a*^	Amplicon size (bp)
*pgdX*	PgdX F	GCACTCGTCAGGGTGATAAA	109
	PgdX R	GATGAGCAAGTTGGAGTGAATG	
	PgdX Probe	FAM-TGATCGTGTTCTCATTACCGGGCG-QSY	
*luxS*	LuxS F	GAAGTGTCTGAGGCTTGGTTAG	103
	LuxS R	CCGTATAGCTTCCGCATTGATAG	
	LuxS probe	FAM-GGTGTACAAGATCAAGCTTCTATTCCCG-QSY	
*dps*	Dps F	GGGCTACCACTGGAACATTA	104
	Dps R	GTTCAGCCACCTCATCTACTC	
	Dps Probe	FAM-TGCGTGTAATGCAAAGAAGTTTACGCC-QSY	
*hktE*	HktE F	CAACAACCAGACTTTGCTGAAC	99
	HktE R	ACGAGGTTGGCTGAAGTAATC	
	HktE Probe	FAM-TCGTATCAACGGAGATGCAGCACA-QSY	
*omp26*	Omp26 F	ACCGCACTTGCTTTAGGTATT	104
	Omp26 R	GCGATCTGGGTGATGTTGAA	
	Omp26 Probe	JOE-TTGCTTCAGGCTATGCTTCCGCT-Zen-BHQ	

a FAM, 6-carboxyfluorescein; JOE, 6-carboxy-4′,5′-dichloro-2′,7′-dimethoxyfluorescein; BHQ, black hole quencher.

### Statistical analyses.

Data were analyzed by one-way analysis of variance (ANOVA) with Tukey’s multiple-comparison test for data sets with normal distribution; for nonparametric data sets, data were analyzed using Kruskal-Wallis analysis with Dunn’s multiple comparison. Specifics regarding statistical methods for individual experiments are provided in the relevant figure legends. *P* values of ≤0.05 were considered statistically significant.

10.1128/mSphere.00847-21.4 FIG S4 Weight of mice 24 h after infection with NTHi or vehicle control. All comparisons of weight were between the vehicle control group (PBS) and the infected group within the same treatment group, air or smoke. Data are means and SD (*n* = 6). Statistical significance was assessed by paired *t* test analysis. Download FIG S4, TIF file, 8.2 MB. Copyright © 2022 Hunt et al. 2022 Hunt et al. https://creativecommons.org/licenses/by/4.0/ This content is distributed under the terms of the Creative Commons Attribution 4.0 International license.

## References

[1] Ekpu VU, Brown AK. 2015. The economic impact of smoking and of reducing smoking prevalence: review of evidence. Tob Use Insights 8:1–35. doi:10.4137/TUI.S15628. 26242225 PMC4502793

[2] Arcavi L, Benowitz NL. 2004. Cigarette smoking and infection. Arch Intern Med 164:2206–2216. doi:10.1001/archinte.164.20.2206.c. 15534156

[3] Hong MJ, Gu BH, Madison MC, Landers C, Tung HY, Kim M, Yuan X, You R, Machado AA, Gilbert BE, Soroosh P, Elloso M, Song L, Chen M, Corry DB, Diehl G, Kheradmand F. 2018. Protective role of gammadelta T cells in cigarette smoke and influenza infection. Mucosal Immunol 11:894–908. doi:10.1038/mi.2017.93. 29091081 PMC5930147

[4] Lawrence H, Hunter A, Murray R, Lim WS, McKeever T. 2019. Cigarette smoking and the occurrence of influenza—systematic review. J Infect 79:401–406. doi:10.1016/j.jinf.2019.08.014. 31465780

[5] Duell BL, Su YC, Riesbeck K. 2016. Host-pathogen interactions of nontypeable Haemophilus influenzae: from commensal to pathogen. FEBS Lett 590:3840–3853. doi:10.1002/1873-3468.12351. 27508518

[6] Erwin AL, Smith AL. 2007. Nontypeable *Haemophilus influenzae*: understanding virulence and commensal behavior. Trends Microbiol 15:355–362. doi:10.1016/j.tim.2007.06.004. 17600718

[7] Sriram KB, Cox AJ, Clancy RL, Slack MPE, Cripps AW. 2018. Nontypeable Haemophilus influenzae and chronic obstructive pulmonary disease: a review for clinicians. Crit Rev Microbiol 44:125–142. doi:10.1080/1040841X.2017.1329274. 28539074

[8] Murphy TF, Kirkham C, Sethi S, Lesse A. 2005. Expression of a peroxiredoxin-glutaredoxin by *Haemophilus influenzae* in biofilms and during human respiratory tract infection. FEMS Immunol Med Microbiol 44:81–89. doi:10.1016/j.femsim.2004.12.008. 15780580

[9] Ahearn CP, Gallo MC, Murphy TF. 2017. Insights on persistent airway infection by non-typeable Haemophilus influenzae in chronic obstructive pulmonary disease. Pathog Dis 75:ftx042. doi:10.1093/femspd/ftx042. PMC5437125 28449098

[10] Freijd A, Bygdeman S, Rynnel-Dagoo B. 1984. The nasopharyngeal microflora of otitis-prone children, with emphasis on H. influenzae. Acta Otolaryngol 97:117–126. doi:10.3109/00016488409130971. 6417970

[11] Kilian M, Heine-Jensen J, Bulow P. 1972. *Haemophilus* in the upper respiratory tract of children. A bacteriological, serological and clinical investigation. Acta Pathol Microbiol Scand B Microbiol Immunol 80:571–578. doi:10.1111/j.1699-0463.1972.tb00181.x. 4566182

[12] Bogaert D, Keijser B, Huse S, Rossen J, Veenhoven R, van Gils E, Bruin J, Montijn R, Bonten M, Sanders E. 2011. Variability and diversity of nasopharyngeal microbiota in children: a metagenomic analysis. PLoS One 6:e17035. doi:10.1371/journal.pone.0017035. 21386965 PMC3046172

[13] Hu YL, Lee PI, Hsueh PR, Lu CY, Chang LY, Huang LM, Chang TH, Chen JM. 2021. Predominant role of Haemophilus influenzae in the association of conjunctivitis, acute otitis media and acute bacterial paranasal sinusitis in children. Sci Rep 11:11. doi:10.1038/s41598-020-79680-6. 33420151 PMC7794412

[14] Swords WE. 2012. Nontypeable Haemophilus influenzae biofilms: role in chronic airway infections. Front Cell Infect Microbiol 2:97. doi:10.3389/fcimb.2012.00097. 22919686 PMC3417564

[15] Reimche JL, Kirse DJ, Whigham AS, Swords WE. 2017. Resistance of non-typeable Haemophilus influenzae biofilms is independent of biofilm size. Pathog Dis 75:ftw112. doi:10.1093/femspd/ftw112. 27956464 PMC5353992

[16] Starner TD, Zhang N, Kim G, Apicella MA, McCray PB, Jr. 2006. *Haemophilus influenzae* forms biofilms on airway epithelia: implications in cystic fibrosis. Am J Respir Crit Care Med 174:213–220. doi:10.1164/rccm.200509-1459OC. 16675778 PMC2662906

[17] Murphy TF, Kirkham C. 2002. Biofilm formation by nontypeable *Haemophilus influenzae*: strain variability, outer membrane antigen expression and role of pili. BMC Microbiol 2:7. doi:10.1186/1471-2180-2-7. 11960553 PMC113772

[18] Silva MD, Sillankorva S. 2019. Otitis media pathogens—a life entrapped in biofilm communities. Crit Rev Microbiol 45:595–612. doi:10.1080/1040841X.2019.1660616. 31502909

[19] Hall-Stoodley L, Hu FZ, Gieseke A, Nistico L, Nguyen D, Hayes JD, Forbes M, Greenberg DP, Dice B, Burrows A, Wackym P, Stoodley P, Post JC, Ehrlich GD, Kerschner JE. 2006. Direct detection of bacterial biofilms on the middle ear mucosa of children with chronic otitis media. JAMA 296:202–211. doi:10.1001/jama.296.2.202. 16835426 PMC1885379

[20] Hall-Stoodley L, Stoodley P. 2009. Evolving concepts in biofilm infections. Cell Microbiol 11:1034–1043. doi:10.1111/j.1462-5822.2009.01323.x. 19374653

[21] Donlan RM. 2001. Biofilm formation: a clinically relevant microbiological process. Clin Infect Dis 33:1387–1392. doi:10.1086/322972. 11565080

[22] Donlan RM. 2002. Biofilms: microbial life on surfaces. Emerg Infect Dis 8:881–890. doi:10.3201/eid0809.020063. 12194761 PMC2732559

[23] Yan J, Bassler BL. 2019. Surviving as a community: antibiotic tolerance and persistence in bacterial biofilms. Cell Host Microbe 26:15–21. doi:10.1016/j.chom.2019.06.002. 31295420 PMC6629468

[24] Bjarnsholt T. 2013. The role of bacterial biofilms in chronic infections. APMIS Suppl 121:1–58. doi:10.1111/apm.12099. 23635385

[25] Hoiby N, Bjarnsholt T, Givskov M, Molin S, Ciofu O. 2010. Antibiotic resistance of bacterial biofilms. Int J Antimicrob Agents 35:322–332. doi:10.1016/j.ijantimicag.2009.12.011. 20149602

[26] Hong W, Juneau R, Pang B, Swords WE. 2009. Survival of bacterial biofilms within neutrophil extracellular traps promotes nontypeable *Haemophilus influenzae* persistence in the chinchilla model for otitis media. J Innate Immun 1:215–224. doi:10.1159/000205937. 20375579 PMC6951045

[27] Rosales C. 2020. Neutrophils at the crossroads of innate and adaptive immunity. J Leukoc Biol 108:377–396. doi:10.1002/JLB.4MIR0220-574RR. 32202340

[28] Sultana S, Foti A, Dahl JU. 2020. Bacterial defense systems against the neutrophilic oxidant hypochlorous acid. Infect Immun 88:e00964-19. doi:10.1128/IAI.00964-19. 32152198 PMC7309615

[29] Harrison A, Bakaletz LO, Munson RS, Jr. 2012. *Haemophilus influenzae* and oxidative stress. Front Cell Infect Microbiol 2:40. doi:10.3389/fcimb.2012.00040. 22919631 PMC3417577

[30] Imlay JA. 2013. The molecular mechanisms and physiological consequences of oxidative stress: lessons from a model bacterium. Nat Rev Microbiol 11:443–454. doi:10.1038/nrmicro3032. 23712352 PMC4018742

[31] Gambino M, Cappitelli F. 2016. Mini-review: Biofilm responses to oxidative stress. Biofouling 32:167–178. doi:10.1080/08927014.2015.1134515. 26901587

[32] Smirnova GV, Oktyabrsky ON. 2005. Glutathione in bacteria. Biochemistry (Mosc) 70:1199–1211. doi:10.1007/s10541-005-0248-3. 16336178

[33] Vergauwen B, Pauwels F, Van Beeumen JJ. 2003. Glutathione and catalase provide overlapping defenses for protection against respiration-generated hydrogen peroxide in Haemophilus influenzae. J Bacteriol 185:5555–5562. doi:10.1128/JB.185.18.5555-5562.2003. 12949108 PMC193741

[34] Vergauwen B, Pauwels F, Vaneechoutte M, Van Beeumen JJ. 2003. Exogenous glutathione completes the defense against oxidative stress in *Haemophilus influenzae*. J Bacteriol 185:1572–1581. doi:10.1128/JB.185.5.1572-1581.2003. 12591874 PMC148052

[35] Vergauwen B, Elegheert J, Dansercoer A, Devreese B, Savvides SN. 2010. Glutathione import in Haemophilus influenzae Rd is primed by the periplasmic heme-binding protein HbpA. Proc Natl Acad Sci USA 107:13270–13275. doi:10.1073/pnas.1005198107. 20628015 PMC2922120

[36] Pauwels F, Vergauwen B, Vanrobaeys F, Devreese B, Van Beeumen JJ. 2003. Purification and characterization of a chimeric enzyme from *Haemophilus influenzae* Rd that exhibits glutathione-dependent peroxidase activity. J Biol Chem 278:16658–16666. doi:10.1074/jbc.M300157200. 12606554

[37] Juneau RA, Pang B, Armbruster CE, Murrah KA, Perez AC, Swords WE. 2015. Peroxiredoxin-glutaredoxin and catalase promote resistance of nontypeable Haemophilus influenzae 86-028NP to oxidants and survival within neutrophil extracellular traps. Infect Immun 83:239–246. doi:10.1128/IAI.02390-14. 25348637 PMC4288874

[38] Vlahos R, Bozinovski S. 2018. Modelling COPD co-morbidities in preclinical models. Respirology 23:1094–1095. doi:10.1111/resp.13416. 30284760

[39] Gotts JE, Chun L, Abbott J, Fang X, Takasaka N, Nishimura SL, Springer ML, Schick SF, Calfee CS, Matthay MA. 2018. Cigarette smoke exposure worsens acute lung injury in antibiotic-treated bacterial pneumonia in mice. Am J Physiol Lung Cell Mol Physiol 315:L25–L40. doi:10.1152/ajplung.00405.2017. 29543040 PMC6087899

[40] Gaschler GJ, Skrtic M, Zavitz CC, Lindahl M, Onnervik PO, Murphy TF, Sethi S, Stampfli MR. 2009. Bacteria challenge in smoke-exposed mice exacerbates inflammation and skews the inflammatory profile. Am J Respir Crit Care Med 179:666–675. doi:10.1164/rccm.200808-1306OC. 19179487

[41] Harrison A, Ray WC, Baker BD, Armbruster DW, Bakaletz LO, Munson RS, Jr. 2007. The OxyR regulon in nontypeable *Haemophilus influenzae*. J Bacteriol 189:1004–1012. doi:10.1128/JB.01040-06. 17142400 PMC1797302

[42] Poole LB. 2015. The basics of thiols and cysteines in redox biology and chemistry. Free Radic Biol Med 80:148–157. doi:10.1016/j.freeradbiomed.2014.11.013. 25433365 PMC4355186

[43] Qian J, Klomsiri C, Wright MW, King SB, Tsang AW, Poole LB, Furdui CM. 2011. Simple synthesis of 1,3-cyclopentanedione derived probes for labeling sulfenic acid proteins. Chem Commun (Camb) 47:9203–9205. doi:10.1039/c1cc12127h. 21738918 PMC3587177

[44] Poole LB. 2008. Measurement of protein sulfenic acid content. Curr Protoc Toxicol Chapter 17:Unit 17.2. doi:10.1002/0471140856.tx1702s38. PMC3943243 20963754

[45] Claiborne A, Yeh JI, Mallett TC, Luba J, Crane EJ, III, Charrier V, Parsonage D. 1999. Protein-sulfenic acids: diverse roles for an unlikely player in enzyme catalysis and redox regulation. Biochemistry 38:15407–15416. doi:10.1021/bi992025k. 10569923

[46] Poole LB, Karplus PA, Claiborne A. 2004. Protein sulfenic acids in redox signaling. Annu Rev Pharmacol Toxicol 44:325–347. doi:10.1146/annurev.pharmtox.44.101802.121735. 14744249

[47] Hunt BC, Stanford D, Xu X, Li J, Gaggar A, Rowe SM, Raju SV, Swords WE. 2020. Haemophilus influenzae persists in biofilm communities in a smoke-exposed ferret model of COPD. ERJ Open Res 6:00200-2020. doi:10.1183/23120541.00200-2020. 32802827 PMC7418822

[48] King L, Pang B, Perez AC, Reimche JL, Kirse DJ, Whigham AS, Evans AK, Swords WE. 2013. Observation of viable nontypeable Haemophilus influenzae bacteria within neutrophil extracellular traps in clinical samples from chronic otitis media. Otolaryngology 3:45.

[49] Juneau RA, Pang B, Weimer KE, Armbruster CE, Swords WE. 2011. Nontypeable *Haemophilus influenzae* initiates formation of neutrophil extracellular traps. Infect Immun 79:431–438. doi:10.1128/IAI.00660-10. 20956567 PMC3019868

[50] Jurcisek JA, Bakaletz LO. 2007. Biofilms formed by nontypeable *Haemophilus influenzae* in vivo contain both double-stranded DNA and type IV pilin protein. J Bacteriol 189:3868–3875. doi:10.1128/JB.01935-06. 17322318 PMC1913342

[51] Jurcisek JA, Bookwalter J, Baker B, Fernandez S, Novotny LA, Munson RS, Jr, Bakaletz LO. 2007. The PilA protein of nontypeable *Haemophilus influenzae* plays a role in biofilm formation, adherence to epithelial cells and colonization of the mammalian upper respiratory tract. Mol Microbiol 65:1288–1299. doi:10.1111/j.1365-2958.2007.05864.x. 17645732

[52] West-Barnette S, Rockel A, Swords WE. 2006. Biofilm growth increases phosphorylcholine content and decreases potency of nontypeable Haemophilus influenzae endotoxins. Infect Immun 74:1828–1836. doi:10.1128/IAI.74.3.1828-1836.2006. 16495557 PMC1418622

[53] Swords WE, Moore ML, Godzicki L, Bukofzer G, Mitten MJ, VonCannon J. 2004. Sialylation of lipooligosaccharides promotes biofilm formation by nontypeable Haemophilus influenzae. Infect Immun 72:106–113. doi:10.1128/IAI.72.1.106-113.2004. 14688087 PMC343998

[54] Hong W, Pang B, West-Barnette S, Swords WE. 2007. Phosphorylcholine expression by nontypeable Haemophilus influenzae correlates with maturation of biofilm communities in vitro and in vivo. J Bacteriol 189:8300–8307. doi:10.1128/JB.00532-07. 17573475 PMC2168690

[55] Hong W, Mason K, Jurcisek J, Novotny L, Bakaletz LO, Swords WE. 2007. Phosphorylcholine decreases early inflammation and promotes the establishment of stable biofilm communities of nontypeable Haemophilus influenzae strain 86-028NP in a chinchilla model of otitis media. Infect Immun 75:958–965. doi:10.1128/IAI.01691-06. 17130253 PMC1828519

[56] Jurcisek JA, Greiner L, Watanabe H, Zaleski A, Apicella MA, Bakaletz LO. 2005. Role of sialic acid and complex carbohydrate biosynthesis in biofilm formation by nontypeable *Haemophilus influenzae* in the chinchilla middle ear. Infect Immun 73:3210–3218. doi:10.1128/IAI.73.6.3210-3218.2005. 15908345 PMC1111813

[57] Armbruster CE, Hong W, Pang B, Dew KE, Juneau RA, Byrd MS, Love CF, Kock ND, Swords WE. 2009. LuxS promotes biofilm maturation and persistence of nontypeable haemophilus influenzae in vivo via modulation of lipooligosaccharides on the bacterial surface. Infect Immun 77:4081–4091. doi:10.1128/IAI.00320-09. 19564381 PMC2738029

[58] Armbruster CE, Pang B, Murrah K, Juneau RA, Perez AC, Weimer KE, Swords WE. 2011. RbsB (NTHI_0632) mediates quorum signal uptake in nontypeable Haemophilus influenzae strain 86-028NP. Mol Microbiol 82:836–850. doi:10.1111/j.1365-2958.2011.07831.x. 21923771 PMC3609414

[59] Pang B, Armbruster CE, Foster G, Learman BS, Gandhi U, Swords WE. 2018. Autoinducer 2 (AI-2) production by nontypeable Haemophilus influenzae 86-028NP promotes expression of a predicted glycosyltransferase that is a determinant of biofilm maturation, prevention of dispersal, and persistence in vivo. Infect Immun 86. doi:10.1128/IAI.00506-18. PMC6246909 30249749

[60] Pang B, Hong W, Kock ND, Swords WE. 2012. Dps promotes survival of nontypeable *Haemophilus influenzae* in biofilm communities in vitro and resistance to clearance in vivo. Front Cell Infect Microbiol 2:1–10. doi:10.3389/fcimb.2012.00058. 22919649 PMC3417516

[61] Pang B, Hong W, Kock ND, Swords WE. 2012. Dps promotes survival of nontypeable Haemophilus influenzae in biofilm communities in vitro and resistance to clearance in vivo. Front Cell Infect Microbiol 2:58. doi:10.3389/fcimb.2012.00058. 22919649 PMC3417516

[62] Zhang L, Xie J, Patel M, Bakhtyar A, Ehrlich GD, Ahmed A, Earl J, Marrs CF, Clemans D, Murphy TF, Gilsdorf JR. 2012. Nontypeable Haemophilus influenzae genetic islands associated with chronic pulmonary infection. PLoS One 7:e44730. doi:10.1371/journal.pone.0044730. 22970300 PMC3435294

[63] Pang B, Winn D, Johnson R, Hong W, West-Barnette S, Kock N, Swords WE. 2008. Lipooligosaccharides containing phosphorylcholine delay pulmonary clearance of nontypeable Haemophilus influenzae. Infect Immun 76:2037–2043. doi:10.1128/IAI.01716-07. 18347044 PMC2346676

[64] Mitchell MA, Skowronek K, Kauc L, Goodgal SH. 1991. Electroporation of *Haemophilus influenzae* is effective for transformation of plasmid but not chromosomal DNA. Nucleic Acids Res 19:3625–3628. doi:10.1093/nar/19.13.3625. 1852608 PMC328389

[65] Yao H, Edirisinghe I, Rajendrasozhan S, Yang SR, Caito S, Adenuga D, Rahman I. 2008. Cigarette smoke-mediated inflammatory and oxidative responses are strain-dependent in mice. Am J Physiol Lung Cell Mol Physiol 294:L1174–L1186. doi:10.1152/ajplung.00439.2007. 18375740

[66] Vecchio D, Arezzini B, Pecorelli A, Valacchi G, Martorana PA, Gardi C. 2010. Reactivity of mouse alveolar macrophages to cigarette smoke is strain dependent. Am J Physiol Lung Cell Mol Physiol 298:L704–L713. doi:10.1152/ajplung.00013.2009. 20154225

[67] Bartalesi B, Cavarra E, Fineschi S, Lucattelli M, Lunghi B, Martorana PA, Lungarella G. 2005. Different lung responses to cigarette smoke in two strains of mice sensitive to oxidants. Eur Respir J 25:15–22. doi:10.1183/09031936.04.00067204. 15640318

[68] Cavarra E, Fardin P, Fineschi S, Ricciardi A, De Cunto G, Sallustio F, Zorzetto M, Luisetti M, Pfeffer U, Lungarella G, Varesio L. 2009. Early response of gene clusters is associated with mouse lung resistance or sensitivity to cigarette smoke. Am J Physiol Lung Cell Mol Physiol 296:L418–L429. doi:10.1152/ajplung.90382.2008. 19118092

[69] Itoh M, Tsuji T, Nakamura H, Yamaguchi K, Fuchikami J, Takahashi M, Morozumi Y, Aoshiba K. 2014. Systemic effects of acute cigarette smoke exposure in mice. Inhal Toxicol 26:464–473. doi:10.3109/08958378.2014.917346. 24932561

[70] Vlahos R, Bozinovski S. 2015. Preclinical murine models of chronic obstructive pulmonary disease. Eur J Pharmacol 759:265–271. doi:10.1016/j.ejphar.2015.03.029. 25818750

[71] Hartmann R, Jeckel H, Jelli E, Singh PK, Vaidya S, Bayer M, Rode DKH, Vidakovic L, Diaz-Pascual F, Fong JCN, Dragos A, Lamprecht O, Thoming JG, Netter N, Haussler S, Nadell CD, Sourjik V, Kovacs AT, Yildiz FH, Drescher K. 2021. Quantitative image analysis of microbial communities with BiofilmQ. Nat Microbiol 6:151–156. doi:10.1038/s41564-020-00817-4. 33398098 PMC7840502

[72] Schindelin J, Arganda-Carreras I, Frise E, Kaynig V, Longair M, Pietzsch T, Preibisch S, Rueden C, Saalfeld S, Schmid B, Tinevez JY, White DJ, Hartenstein V, Eliceiri K, Tomancak P, Cardona A. 2012. Fiji: an open-source platform for biological-image analysis. Nat Methods 9:676–682. doi:10.1038/nmeth.2019. 22743772 PMC3855844

[73] Chang AC, Cohen SN. 1978. Construction and characterization of amplifiable multicopy DNA cloning vehicles derived from the P15A cryptic miniplasmid. J Bacteriol 134:1141–1156. doi:10.1128/jb.134.3.1141-1156.1978. 149110 PMC222365

